# MiR-338-3p Inhibits Hepatocarcinoma Cells and Sensitizes These Cells to Sorafenib by Targeting Hypoxia-Induced Factor 1α

**DOI:** 10.1371/journal.pone.0115565

**Published:** 2014-12-22

**Authors:** Haitao Xu, Liang Zhao, Qiuju Fang, Jianmin Sun, Songyan Zhang, Chao Zhan, Shujie Liu, Yubao Zhang

**Affiliations:** 1 Department of Hepatopancreatobiliary Surgery, Harbin Medical University Cancer Hospital, Harbin, Heilongjiang, P.R. China; 2 Department of Internal Medicine, The Heilongjiang Provincial Hospital, Harbin, Heilongjiang, P.R. China; 3 Department of Operations, Harbin Medical University Cancer Hospital, Harbin, Heilongjiang, P.R. China; Thomas Jefferson University, United States of America

## Abstract

Hypoxia is a common feature of solid tumors and an important contributor to anti-tumor drug resistance. Hypoxia inducible factor-1 (HIF-1) is one of the key mediators of the hypoxia signaling pathway, and was recently proven to be required for sorafenib resistance in hepatocarcinoma (HCC). MicroRNAs have emerged as important posttranslational regulators in HCC. It was reported that miR-338-3p levels are associated with clinical aggressiveness of HCC. However, the roles of miR-338-3p in HCC disease and resistance to its therapeutic drugs are unknown. In this study, we found that miR-338-3p was frequently down-regulated in 14 HCC clinical samples and five cell lines. Overexpression of miR-338-3p inhibited HIF-1α 3′-UTR luciferase activity and HIF-1α protein levels in HepG2, SMMC-7721, and Huh7 cells. miR-338-3p significantly reduced cell viability and induced cell apoptosis of HCC cells. Additionally, HIF-1α overexpression rescued and HIF-1α knock-down abrogated the anti-HCC activity of miR-338-3p. Furthermore, miR-338-3p sensitized HCC cells to sorafenib *in vitro* and in a HCC subcutaneous nude mice tumor model by inhibiting HIF-1α. Collectively, miR-338-3p inhibits HCC tumor growth and sensitizes HCC cells to sorafenib by down-regulating HIF-1α. Our data indicate that miR-338-3p could be a potential candidate for HCC therapeutics.

## Introduction

Hepatocarcinoma (HCC) is one of the most common human malignancies, causing more than 600,000 deaths worldwide each year. Although half of cases and deaths were estimated to occur in China, the incidence is increasing not only in Asia, but also in the USA, Europe, and Africa [1]. Treatment options for HCC include surgical resection, liver transplantation, radioimmunotherapy, and chemotherapy. The choice of treatment depends on the cancer stage, resource availability, and practitioner choices [2]. Chemotherapy is an important therapeutic strategy for patients who are in advanced stages of disease but are not candidates for surgery [3]. Sorafenib, a multi-kinase inhibitor, is the only clinically approved drug for patients with advanced HCC [4]; however, high rates of sorafenib resistance in HCC patients often prevent its long-term efficacy [5]. Therefore, novel targets and approaches are needed to successfully treat this deadly cancer.

Hypoxia is commonly observed in malignant neoplastic tissue as tumors increase in size but lack neurovascularization [6]. Hypoxia-inducible factor (HIF)-1 is a transcription factor that mediates cell adaptive responses to hypoxia by regulating a series of genes implicated in angiogenesis, glucose uptake, metabolism, and cell proliferation [7]. As a consequence of intratumoral hypoxia, HIF-1 was found to be overexpressed and play important roles in the pathogenesis and pathophysiology of HCC [8]–[10]. Recent studies suggested that tumor hypoxia results in chemotherapy resistance, and that HIF-1 plays a critical role in hypoxia-induced chemoresistance. [10]–[12]. As a promising therapeutic target for HCC, HIF-1 when inhibited has been shown to suppress tumor growth and to reverse chemoresistance [13]–[15]. HIF-1 is a heterodimer protein composed of an oxygen-sensitive HIF-1α subunit and a constitutively expressed HIF-1β subunit [16]. Although oxygen-dependent post-translational modification is the primary mechanism of HIF-1α accumulation, HIF-1α can also be transcriptionally and translationally regulated by signaling molecules such as growth factors, cytokines and microRNAs [17].

MicroRNA is a class of small, endogenous, non-coding RNA molecules that control gene expression by targeting mRNAs for cleavage or repression of translation. [18] miRNAs are differentially expressed in normal tissues and cancers, and contribute to cancer development and progression [19]. In this study, we found that miR-338-3p directly targeted HIF-1α and suppressed the HIF signaling pathway. We examined the tumor suppressor properties of miR-338-3p in HCC cells and in nude mice. Furthermore, our data showed that miR-338-3p potentiated growth inhibitory function of sorafenib in HCC.

## Materials and Methods

### Samples

Study involving human participants was approved by the institutional review board at Harbin Medical University. Written consent was given by all of the patients according to the Declaration of Helsinki and documented. None of the patients in the study received chemotherapy or radiation therapy before surgery.

### Cell lines

The human hepatoma cell lines, HepG2, SMMC-7721, BEK-7402, Hep3B, and Huh-7, and the liver cell line L02 were purchased from the cell bank of type culture collection at the Chinese Academy of Sciences (Shanghai, China). Sorafenib (sc-220125A) was purchased from Santa Cruz Biotechnology (Santa Cruz, CA) and dissolved in DMSO. The final DMSO concentration was lower than 0.1%.

### Hypoxia treatment

Hypoxia treatment was conducted as previously described [20]. Briefly, cells were placed in a sealed hypoxia chamber equilibrated with certified gas containing 1% O_2_, 5% CO_2_, and 94% N_2_.

### RNA extraction and real time PCR (RT-PCR)

Total miRNA was extracted using the TRIzol reagent (Invitrogen, Carlsbad, CA). Complementary DNA was synthesized using the Taqman miRNA reverse transcription kit (Invitrogen). The expression levels of miR-338-3p were quantified using TaqMan miRNA assay kit (Applied Biosystems, Foster City, CA). For mRNA expression analysis, first-strand cDNA synthesis was performed using the Superscript III reverse transcription system (Invitrogen). RT-PCR was performed in triplicate in the ABI 7500HT Fast Real-Time PCR System (Applied Biosystems). The sequences of the primers were as follows: *HIF1A* forward GAAAGCGCAAGTCTTCAAAG; reverse TGGGTAGGAGATGGAGATGC; *MDR1* forward CTGGTTTGATGTGCACGATGTTGG, reverse TGCCAAGACCTCTTCAGCTACTG; *VEGF* forward TGCAGATTATGCGGATCAAACC, reverse TGCATTCACATTTGTTGTGCTGTAG; *GLUT-1* forward ATACTCATGACCATCGCGCTAG, reverse AAAGAAGGCCACAAAGCCAAAG; *GAPDH* forward TGCACCACCAACTGCTTAGC, reverse CCACCACCCTGTTGCTGTAG.

### miRNA mimics, siRNA and plasmids

miR-338-3p mimic, miR-338-3p inhibitor, miR-338-3p mutant and negative control (NC) were purchased from Shanghai Gene-Pharma Co. (Shanghai, China). HIF1α-siRNA (sc-35561) and control siRNA-A (sc-37007) were purchased from Santa Cruz Biotechnology, Inc. HIF1α plasmid (18949) [21] and hypoxia response element (HRE)-luc pGL vector (26731) [22] were purchased from Addgene (Cambridge, MA). Control pGL vector were obtain from Promega (Madison, WI). The 3′UTR of HIF1A was PCR-amplified from HepG2 cDNA and cloned downstream of the luciferase gene in the pGL vector (Promega). Plasmid, siRNA, and miRNA transfection was performed using Lipofectamine 2000 (Invitrogen). Cells were subjected to functional or mechanistic analyses two days post-transfection. Luciferase activity was measured using the dual-luciferase reporter system (Promega). Renilla activity was used to normalize the relative firefly luciferase values.

### Protein isolation and western blotting

Total proteins were extracted with RIPA buffer containing proteinase/phosphatase inhibitors (Thermo, Cambridge, MA). Proteins were separated on a 10% SDS-PAGE gel, and then transferred onto polyvinylidene difluoride membrane (Millipore, Bedford, MA). The membrane was incubated with one of the following antibodies from Santa Cruz Biotechnology, Inc: anti-HIF-1α (sc-4438 WB), anti-P-gp (sc-55510), anti-VEGF (sc-48835), anti-GLUT-1 (sc-7903), or anti-β-actin (sc-1616).

### Immunofluorescence staining

Cells were fixed in ice-cold acetone for 10 min, then blocked with 1% BSA in PBS for 30 min, and then incubated with anti-HIF-1α antibody (1∶50) for 1 h. Cells were washed in PBS prior to incubation with an Alexa-568-conjugated secondary antibody (1∶50; Vector Lab, Burlingame, CA). Nuclei were stained with DAPI.

### Cell viability assays

Cell viability was measured using MTT assay (Promega) as described previously. [23] Briefly, two days post-transfection, 3000 cells were seeded into wells of 96-well plates, and after 24 h, cells were starved in DMEM +0.1% FBS overnight, then cultured in DMEM +10% FBS under hypoxia for different periods of time up to six days. MTT (20 µl) solution was added for 1 h at 37°C. Absorbance was recorded at 490 nm using a microplate reader (Bio-Rad, Richmond, CA). Each individual experiment was performed in six replicates three times independently.

### Cell apoptosis assay by flow cytometry

Two days post-transfection, cells were cultured under hypoxia for two days, and then stained with FITC-conjugated Annexin V (Clontech, Beijing, China) for apoptotic cells and with PI for necrotic cells. Cells were counted using a LSRII flow cytometer (BD Biosciences, San Jose, CA) and analyzed by Flow Jo.

### Animals and subcutaneous tumor growth

Male athymic BALB/c nude mice (10 weeks old, 20 g) were used in this study. Animal study was approved by the Institutional Animal Care and Use Committee of Harbin Medical University (Protocol: 2012–003). Mice were randomly divided into four groups (n = 8) that received either lentivirus vector, lentivirus vector +sorafenib, lentivirus-miR-338-3p, or lentivirus-miR-338-3p + sorafenib. HepG2 cells were infected with 20 MOI of miR-338-3p-expressing lentivirus or control lentivirus by spin infection for 2 h, followed by incubation at 37°C for 2 h. HepG cells (2×10^6^) in 0.1 ml Hank's balanced salt solution were injected subcutaneously into the right scapular region of nude mice. From the seventh day, mice were administered once daily with sorafenib (orally, 10 mg/kg on days 1–5 of each week). Tumor size was determined every seven days by caliper measurement of two perpendicular diameters of the implants, and animal body weights were recorded every seven days. Tumor-bearing mice were sacrificed on day 35 by decapitation without anesthesia.

### Immunohistochemistry

Tumor sections were immunostained with anti-HIF-1α antibody. Briefly, paraffin-embedded tissues were deparaffinized and rehydrated prior to antibody addition. Anti-HIF-1α antibody was used at a dilution of 1∶500.

### Statistics

All values are expressed as mean ± SEM. Differences between groups were analyzed by one-way ANOVA, followed by Bonferroni post-hoc analyses as appropriate. P<0.05 was considered significant.

## Results

### miR-338-3p expression is significantly reduced in HCC tissues and cell lines

**Figure 1:**
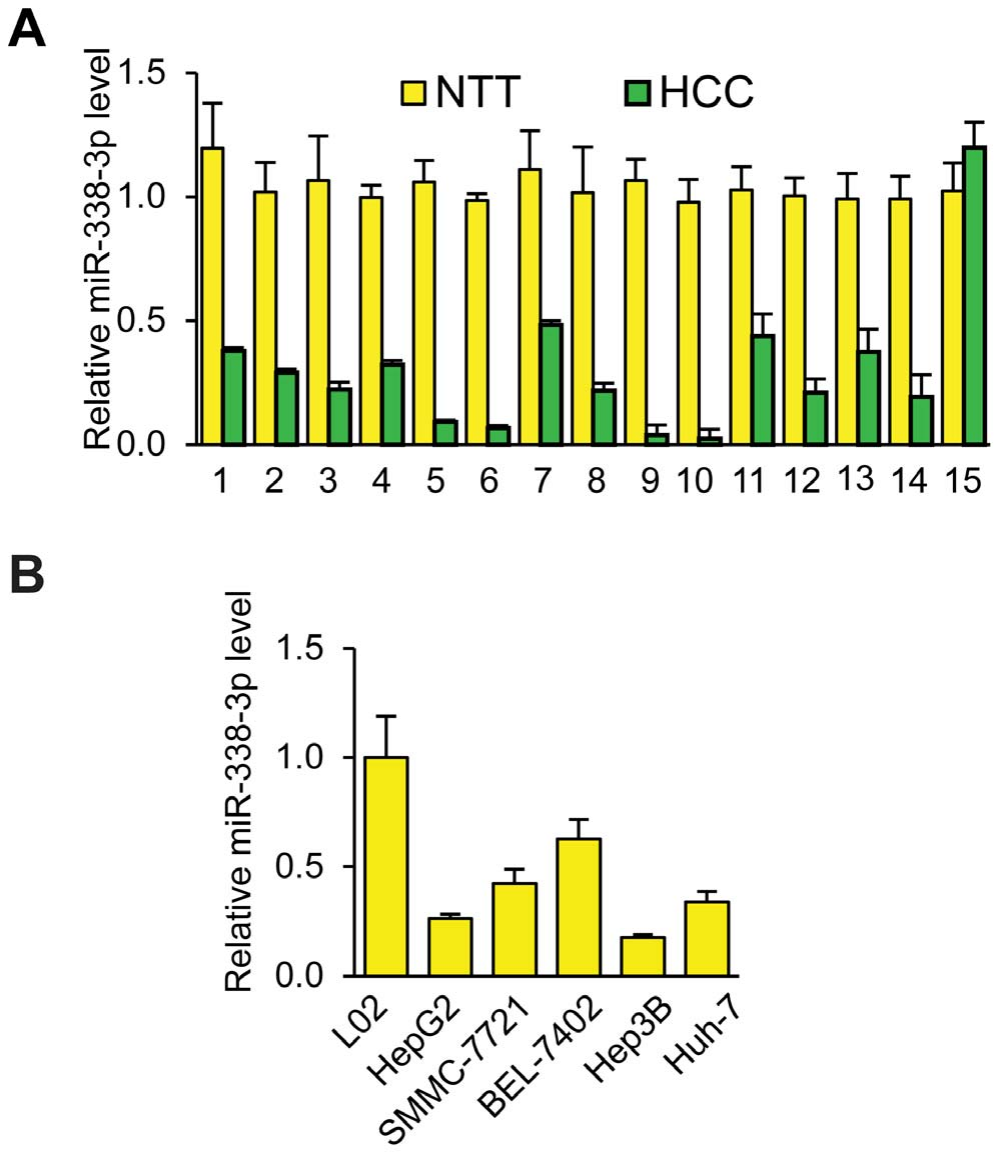
Down-regulation of miR-338-3p in human Hepatocarcinoma (HCC) tissues and cell lines. (A) Real-time PCR analysis of miR-338-3p expression in 15 HCC specimens compared to their pair-matched adjacent non-tumor tissues (NTT). Expression levels were normalized to U6 snRNA and expressed as relative change compared to NTT. (B) Real-time PCR analysis of miR-338-3p expression in the liver cell line L02 and HCC cell lines HepG2, SMMC-7721, BEK-7402, Hep3B, and Huh-7. Expression levels were normalized to U6 snRNA and expressed as relative change compared to L02. Data are shown as mean ± SEM of three independent experiments.

To determine whether miR-338-3p is involved in regulation of human HCC tumorigenesis, we first detected miR-338-3p levels in HCC tumor and adjacent non-tumor tissues, using RT-PCR (n = 15). As shown in Fig. 1A, miR-338-3p expression was significantly less in 14 HCC samples and significantly more in one HCC sample compared to normal adjacent liver tissue. We also analyzed miR-338-3p expression in liver cell line L02 and five human HCC cell lines (HepG2, SMMC-7721, BEK-7402, Hep3B, and Huh-7). Consistently, miR-338-3p was down-regulated in all HCC cell lines examined compared to L02 cells (Fig. 1B). Taken together, these findings suggested that miR-338-3p is down-regulated in human HCC.

### miR-338-3p directly targets HIF-1α

**Figure 2:**
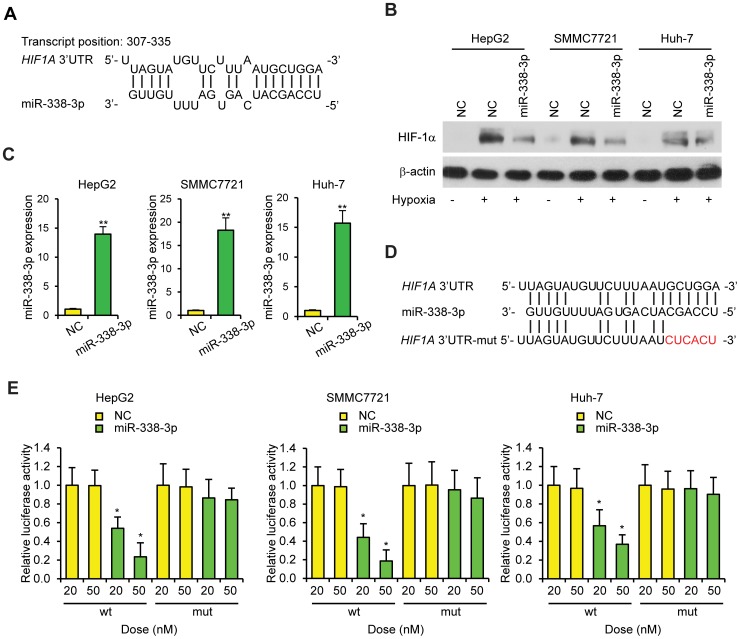
miR-338-3p suppresses HIF-1α expression by directly targeting the *HIF1A* 3′UTR. (A) Predicted miR-338-3p target sequences in the *HIF1A* 3′UTR. (B) Western blot analysis of HIF-1α levels in NC- or miR-338-3p-transfected cells. β-actin was used as loading control. Cells were cultured under hypoxia at two days post-transfection with cells cultured under normoxia as reference, and 24 h later protein levels were analyzed. (C) Relative miR-338-3p levels in miR-338-3p mimic transfected HCC cells. Transcript levels were normalized to *U6* expression. n = 4. (D) Six nucleotides (red) of HIF 3′UTR were mutated to prevent binding with miR-338-3p. (E) Luciferase reporter assay of cells transfected with the wild type (wt) or mutant (mut) *HIF1A* 3′UTR luciferase reporter plasmid with increasing amounts (20 to 50 nM) of negative control miRNA (NC) or miR-338-3p in HCC cells two days post-transfection; n = 4. **p≤0.01 compared to NC group. Data are shown as mean ± SEM of three independent experiments.

Using the DNA Intelligent Analysis -miRPath v2.0 program, we observed that HIF-1α contains conserved miR-338-3p recognition sites in its 3′-UTR (Fig. 2A). [24], [25] To confirm that miR-338-3p regulates HIF-1α expression, we assessed HIF-1α protein levels in HepG2, SMMC7721, and Huh-7 cells expressing ectopic miR-338-3p, using western blot. The results showed that HIF-1α levels, under hypoxia, were consistently reduced by miR-338-3p overexpression in all three types of cell lines (Fig. 2B). Using miRNA-specific RT-PCR, we confirmed that the miR-338-3p level had increased more than 10-fold after transfection (Fig. 2C). To further demonstrate that miR-338-3p directly regulates HIF-1α by interacting with its 3′UTR, we co-transfected the pGL luciferase reporter plasmid harboring the wild type or mutant 3′-UTR of HIF-1α, along with miR-338-3p or NC-miRNA (Fig. 2D). Overexpression of miR-338-3p resulted in significant reduction of HIF1A 3′ UTR firefly luciferase reporter activity containing wild type but not mutant binding sites compared to that of NC-miRNA (Fig. 2E; p≤0.01). We did not observe significant difference in luciferase activity in cells transfected with miR-338-3p inhibitor compared to NC. This may be due to the low endogenous levels of miR-338-3p in HCC cells (S1 Fig.). In summary, these results indicate that HIF-1α is a direct target gene of miR-338-3p in human HCC cells.

### miR-338-3p inhibits HIF signaling pathway

**Figure 3:**
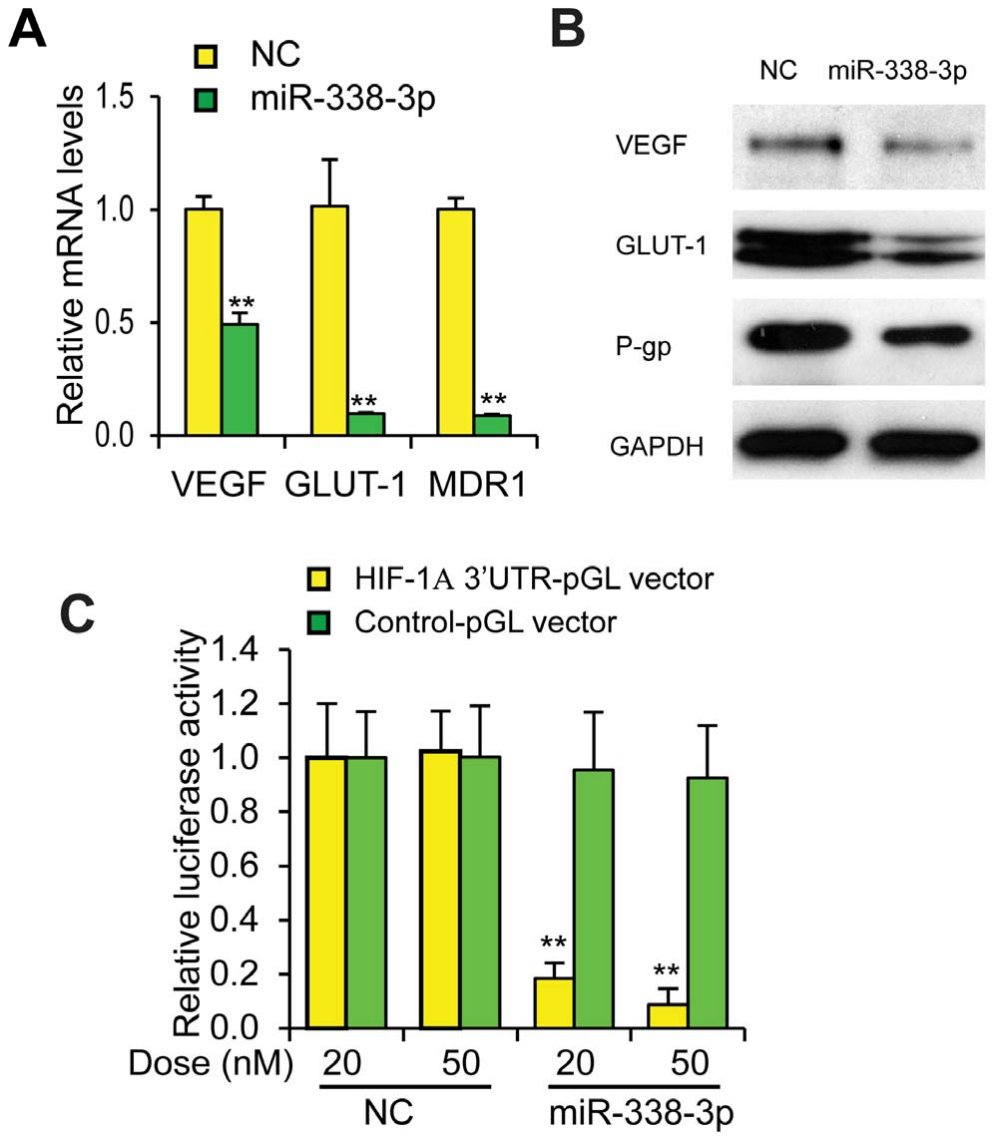
miR-338-3p inhibits the HIF pathway. (A) Effect of miR-338-3p on HIF-1α target genes expression including VEGF, GLUT-1 and MDR1, determined by RT- PCR. HepG2 cells were transfected with 50 µM NC or miR-338-3p for 48 h. RT-PCR was performed 24 h after cells were incubated under hypoxia. n = 3. **p≤0.01 to NC group. (B) Effect of miR-338-3p on VEGF, GLUT-1, and MDR1 expression determined by western blot. (C) Luciferase assay of HepG2 cells co-transfected with the hypoxia response element -luc reporter or control-luc reporter with and increasing doses of NC or miR-338-3p. Cells were incubated under hypoxic conditions for 24 h. Firefly luciferase values were normalized to Renilla luciferase activity; n = 4. Data are shown as mean ± SEM of three independent experiments.

We next used RT-PCR and western blot to examine whether miR-338-3p overexpression results in down-regulation of the HIF signaling pathway. As shown in Fig. 3A and B, miR-338-3p overexpression down-regulated expression of HIF-1 target genes vascular endothelial growth factor (VEGF), glucose transporter 1 (GLUT-1), and multidrug resistance gene (MDR1, produces P-glycoprotein; P-gp) at the transcriptional and the translational levels under hypoxia. Moreover, to determine whether miR-338-3p could affect the transcriptional activity of HIF-1α, we co-transfected HIF-1α luciferase reporter plasmid with miR-338-3p or NC-miRNA into HepG2 cells. As expected, miR-338-3p, in a dose-dependent manner, decreased the relative luciferase activity (Fig. 3C; p≤0.01). Collectively, our data demonstrates the functional link between miR-338-3p and the HIF signaling pathway in human HCC cells.

### miR-338-3p reduces HCC cell viability and promotes cell apoptosis

**Figure 4:**
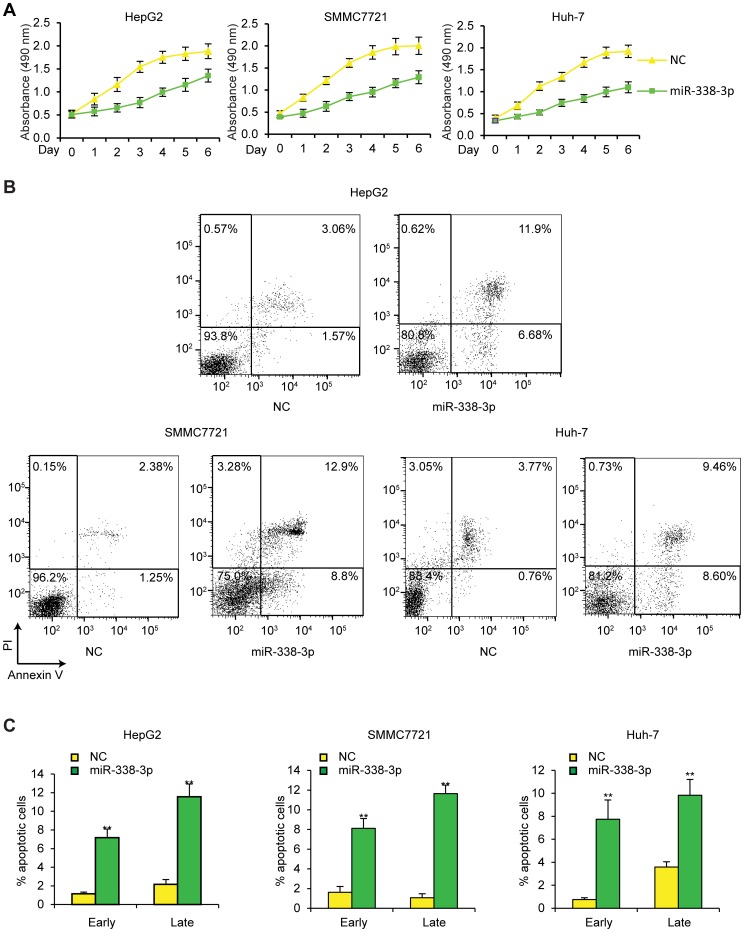
miR-338-3p reduces HCC cell viability and induces cell apoptosis under hypoxia. (A) Cell viability was determined by MTT assays in NC- or miR-338-3p- (50 nM) transfected HCC cells under hypoxic conditions; n = 4. (B) Annexin V/PI double staining showing percentage of early and late apoptotic cells in NC- or miR-338-3p- (50 nM) transfected HCC cells. (C) % apoptotic cells in early and late stage cell growth; n = 4. **p≤0.01 compared to NC-miRNA group. Data are shown as mean ± SEM of three independent experiments.

To establish whether miR-338-3p plays a suppressing role in HCC tumorigenesis, we analyzed cell viability using MTT assay and apoptosis using flow cytometry, in HCC cells transfected with miR-338-3p. As shown in Fig. 4A, ectopic expression of miR-338-3p markedly reduced HCC cell viability in all three types of HCC cell lines. Similarly, miR-338-3p significantly increased early and late apoptotic cell populations of human HCC cells (Figs. 4B and 4C; p≤0.01). The effect of miR-338-3p on HCC cell growth was also investigated under normoxia condition. The results showed that the inhibitory effect of miR-338-3p on HCC cells were more significant under hypoxia than under normoxia (S2 Fig.). Taken together, these results suggested anti-cell growth properties of miR-338-3p in HCC cells.

### The inhibitory effect of miR-338-3p on HCC is mediated by down-regulating HIF-1α

**Figure 5:**
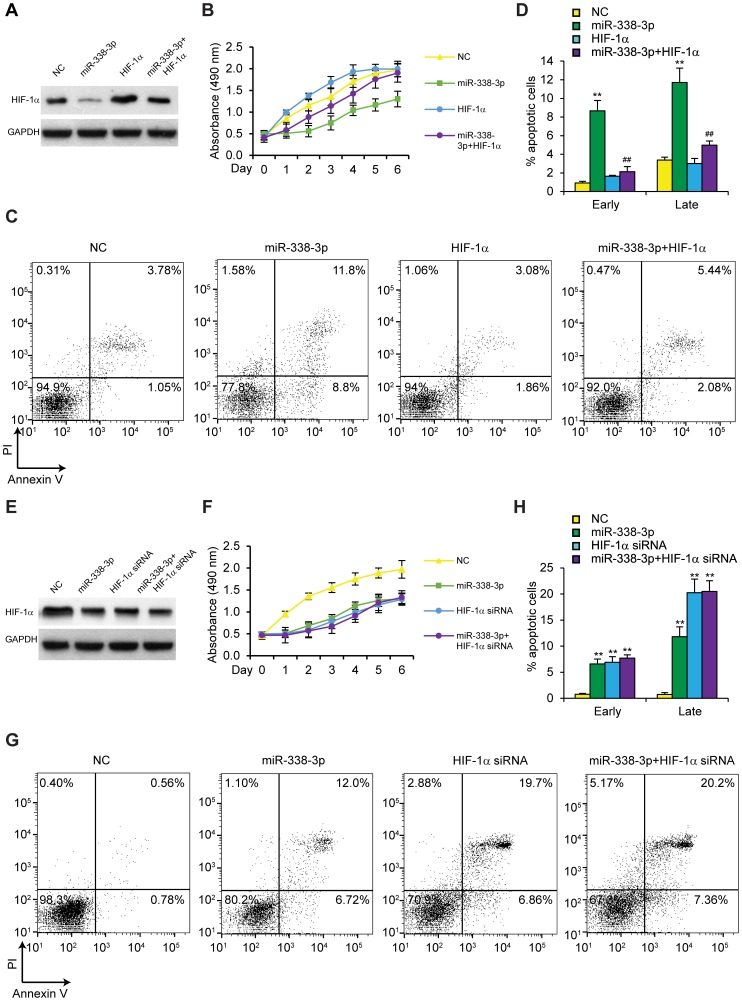
Inhibitory effect of miR-338-3p on HCC cells is mediated by down-regulating HIF-1α. (A) HIF-1α levels in HepG2 cells transfected with miR-338-3p and/or HIF-1α determined by western blot. (B) Cell viability and (C-D) % apoptotic in HepG2 cells transfected with miR-338-3p and/or HIF-1α plasmid under hypoxic conditions; n = 4. (E) HIF-1α levels in HepG2 cells transfected with miR-338-3p and/or HIF-1A siRNA determined by western blot. (F) Cell viability and (G-H) % apoptotic in HepG2 cells transfected with miR-338-3p and/or HIF-1A siRNA under hypoxia; n = 4. Cell apoptosis was assessed after two days of culture under hypoxic conditions. **p≤0.01 compared to NC group. ## p≤0.01 compared to miR-338-3p only group. Data are shown as mean ± SEM of three independent experiments.

To explore the functional significance of HIF-1α in the inhibitory effects of miR-338-3p on HCC tumorigenesis, we overexpressed HIF-1α in miR-338-3p-transfected HepG2 cells and determined whether HIF-1α can reverse miR-338-3p-mediated regulation of cell viability and apoptosis using western blot, MTT assay and flow cytometry. As shown in Fig. 5A and 5B, re-introduction of HIF-1α rescued HIF-1α protein levels downregulated by miR-338-3p and abrogated the inhibitory effect of miR-338-3p on cell viability. Additionally, apoptosis induced by miR-338-3p was also significantly attenuated by HIF-1α overexpression (Fig. 5C-D; p≤0.01). Consistent with the results of our HIF-1α overexpression study, down-regulation of HIF-1α, using HIF1A siRNA, significantly decreased HIF-1α protein levels, reduced HepG2 cell viability and induced cell apoptosis, whereas miR-338-3p did not show further effects when co-transfected with HIF1A siRNA (Figs. 5E-5H; p≤0.01). These results indicate that miR-338-3p elicits anti-HCC effects by targeting HIF-1α.

### miR-338-3p sensitizes HCC cells to sorafenib

**Figure 6:**
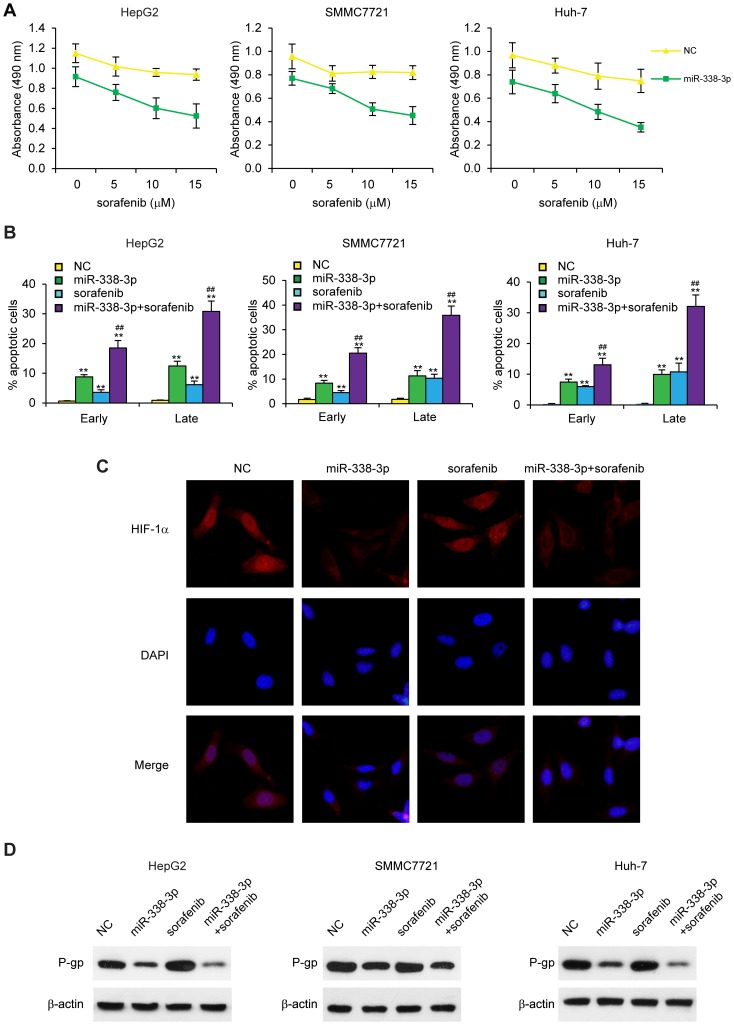
miR-338-3p sensitizes HCC cells to sorafenib treatment. (A) Cell viability was determined in NC- or miR-338-3p- (50 nM) transfected cells at two days after sorafenib (0, 5, 10. 15 µM) treatment under hypoxia; n = 4. (B) % apoptotic cells assessed two days after sorafenib (15 µM) treatment under hypoxia; n = 4. (C) Cytoplasmic and nuclear expression of HIF-1α in NC- or miR-338-3p-transfected (50 nM) HepG2 cells treated with or without sorafenib (15 µM), as detected by immunofluorescence staining. Cells were incubated under hypoxia for 24 h. Red is HIF-1α staining. Blue is the nuclear staining by DAPI. (D) Western blot analysis of P-gp levels in NC- or miR-338-3p-transfected cells with or without sorafenib (15 µM) treatment for two days under hypoxia. β-actin was used as loading control. **p≤0.01 compared to NC group. ## p≤0.01 compared to sorafenib only group. Data are shown as mean ± SEM of three independent experiments.

Because recent studies have reported that inhibition of HIF-1α can overcome hypoxia-mediated sorafenib resistance in HCC [20], we tested whether miR-338-3p could sensitize HCC cells to sorafenib treatment. We treated miR-338-3p-transfected cells and NC cells with sorafenib and measured cell viability. Cell viability (MTT assay) and cell apoptosis (flow cytometry assays showed that non-transfected NC HCC cells are highly resistant to sorafenib under hypoxia and that transfection with miR-338-3p significantly reduced sorafenib resistance (Fig. 6A, B). Next, we investigated the effect of miR-338-3p on HIF-1α expression under hypoxia in HepG2 cells with or without sorafenib treatment. Immunofluorescence staining results revealed that HIF-1α was accumulated into the nuclei in control and sorafenib-treated cells under hypoxia. However, the overall staining and nuclear accumulation of HIF-1α was markedly reduced with miR-338-3p transfection (Fig. 6C). To further elucidate the mechanisms through which miR-338-3p reduces HCC resistance to sorafenib, we tested the effect of miR-338-3p on P-gp gene expression, since inducing P-gp protein expression is considered one of the most important mechanisms of HIF-1α on chemoresistance [10], [26]. The results showed that under hypoxia, P-gp was highly expressed in HCC cells. However, P-gp levels were significantly reduced in miR-338-3p-transfected cells (Fig. 6D). Taken together, our results provide strong evidence that miR-338-3p can antagonize hypoxia-mediated sorafenib resistance by regulating HIF-1α.

### miR-338-3p and sorafenib synergistically inhibit subcutaneous tumor growth

**Figure 7:**
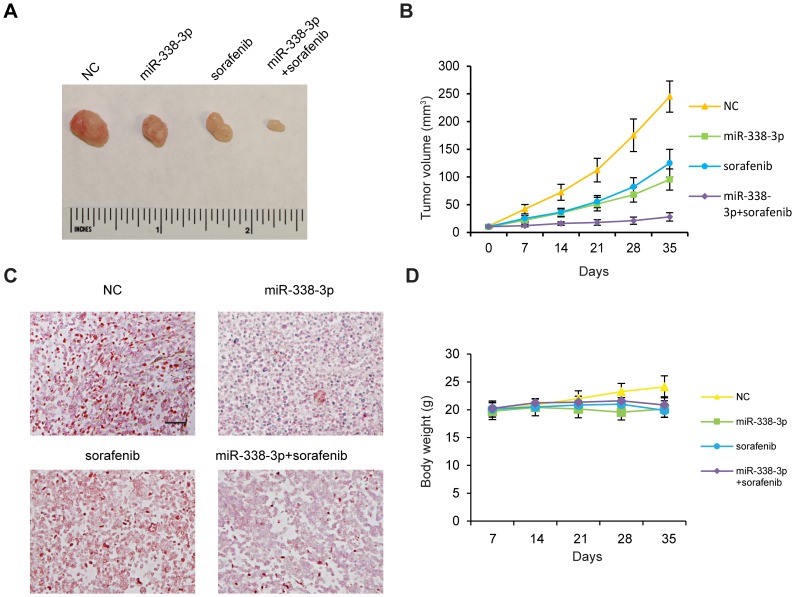
miR-338-3p and sorafenib synergistically inhibited subcutaneous tumor growth. (A) Representative photos of tumor tissues from different treatment groups 35 days post-injection. (B) Mean tumor volumes measured every seven days. (C) Representative photos of sections stained with an anti-HIF-1α antibody; scale bar 0.15 mm. (D) Average mouse body weight. n = 8 in each group. Data are shown as mean ± SEM.

We observed that miR-338-3p sensitized human HCC cells to sorafenib *in vitro*. We then evaluated the ability of miR-338-3p to potentiate the anti-tumor effects of sorafenib in a mouse subcutaneous injection model. Tumors from control mice showed a gradual increase in tumor volume. However, tumors treated with miR-338-3p or sorafenib were smaller than control tumors. Notably, tumors treated with miR-338-3p and sorafenib were significantly smaller than tumors treated with either miR-338-3 or sorafenib alone (Figs. 7A and 7B). Moreover, miR-338-3p down-regulated tumor HIF-1α expression in mice treated with or without sorafenib (Fig. 7C). Mice treated with sorafenib and/or miR-338-3p showed moderate weight loss (Fig. 7D). In summary, combined treatment with sorafenib and miR-338-3p exerted a more potent anti-tumor growth effect than either alone.

## Discussion

Growing evidence indicates that miRNAs hold great promise for novel therapeutic approaches for treating human cancers. Deregulation of miR-338-3p has been reported for many different cancer types. Even though recent evidence indicates the inhibitory effect of miR-338-3p on human cancers, such as colorectal [27], neuroblastoma [28], gastric [29], and osteosarcoma [30], there is little knowledge about miR-338-3p and its targets in HCC.

Our study found that miR-338-3p expression is markedly down-regulated in HCC patient samples and HCC cell lines as compared to normal liver cells. Furthermore, miR-338-3p could reduce HCC cell viability and promote cell apoptosis by directly binding to the 3′-UTR of HIF-1α. We demonstrated that miR-338-3p can sensitize HCC cells to sorafenib. These findings suggest that miR-338-3p is a potential HCC suppressor and plays an important role in preventing HCC drug resistance.

Predicted targets of miR-338-3p are factors involved in many biological processes, such as cell proliferation, differentiation, and cell death, as well as diseases such as Alzheimer's, arthritis, and cancer. Our study identified one key target of miR-338-3p, HIF-1α. HIF-1α is the major transcription factor that is activated in many tumors showing either promoter or suppressor activity. As with most solid tumors, the hypoxic microenvironment exists in HCC as a result of a shortage of blood circulation and high proliferation of tumor cells. Hypoxia enhances proliferation [31], [32] and suppresses differentiation [33] and apoptosis [34] of HCC, thereby resulting in tumor malignancy. HCC cells survive and proliferate in a hypoxic microenvironment mainly by stabilizing and activating HIFs. The active HIFs can induce expression of various genes controlling angiogenesis, glucose metabolism, cell survival, and tumor spread [35], [36]. Our results showed that miR-338-3p inhibits cell viability and induces cell apoptosis by directly targeting HIF-1α. These results support the possibility that HIF-1α functions as a tumor promoter in the liver, and indicates potential applications for miR-338-3p in anticancer therapy. Previous studies have shown that other cell regulatory elements such as cyclin D1 [37] and *smoothened* [38] also are targets of miR-338-3p that are aberrantly expressed due to downregulated miR-338-3p expression in HCC. Undoubtedly, regulation of such other targets may contribute to the inhibitory effects of miR-338-3p on HCC. However, considering our observation that HIF-1α overexpression rescued the cell from the anti-HCC activity of miR-338-3p, it is likely that regulation of HIF-1α by miR-338-3p is a key anti-tumor aspect in HCC. Our further studies will focus on other targets of miR-338-3p and their specific roles under both normoxic and hypoxic conditions.

Our study found that miR-338-3p overexpression down-regulated expression of VEGF, GLUT-1 and MDR1, which are all known to be regulated by HIF-1 and are important in tumorigenesis [39]–[41]. The delivery of nutrients to tumor cells is essential to their survival and hence angiogenesis is essential to the growing tumor [42], which relies on the expression of angiogenic factors by the cancer cells [43]. On such factor is VEGF. Early studies showed that inhibiting VEGF, tumor angiogenesis and tumor growth become impaired [44], [45]. VEGF expression can be initiated by hypoxia and then contributes significantly to tumor angiogenesis [46], [47]. Additionally, VEGF induces permeabilization of blood vessels vesiculo-vacuolar organelle formation, aiding protein transport leading to extravascular fibrin formation. The latter is a cell growth supporting matrix that facilitates stromal cell invasion of the developing tumor [48]–[51]. GLUT-1 is a membrane spanning enzyme that transports glucose across the cell's plasma membrane and is highly expressed in blood vessel endothelium [52].

Transcription of the MDR1 gene expresses P-gp, an energy dependant membrane efflux pump. P-gp can transport a wide range of xenobiotics to sustain non-toxic concentrations in the cytoplasm [53]. Expression of *MDR1* can be found in some normal cell types, however P-gp overexpression correlates with multidrug resistance, and P-gp overexpression occures in many multidrug-resistant cell lines. Exactly how P-gp overexpression is facilitated in cancer is currently not fully understood and appears to be complex [53], [54]. The MDR1 promoter incorporates several transcription factor binding sites, such as SP1, NF-Y, and YB-1 [55] and negative regulation has of MDR1 was demonstrated to be mediated by the p65 subunit of NF-κB with c-fos [56]. To our knowledge, our study is the first to show regulation of P-gp expression by miR-338-3p.

Certain miRNAs and HIF-1α confer drug resistance [57] or sensitivity [58] to cancer cells. We wanted to determine whether miR-338-3p potentiates sensitivity of HCC cells to sorafenib, which is the only drug that currently improves overall survival of HCC patients [59]. A recent study reported that sustained sorafenib treatment leads to decreased microvessel density and increased HIF-1α protein levels and transcriptional activity in HCC [20], consequently leading to sorafenib resistance. Inhibited or silent HIF-1α can increase HCC sensitivity to sorafenib, which provides a rationale for testing combined therapy with miR-338-3p and sorafenib. Our *in vitro* results showed that under hypoxic conditions, HCC cells are highly resistant to sorafenib. However, cells pre-transfected with miR-338-3p can overcome hypoxia-mediated sorafenib resistance. We also found that miR-338-3p combined with sorafenib has synergistic effects against HCC tumor growth *in vivo*. In this regard, the chemosensitizing effect of miR-338-3p is an important feature for its potential therapeutic roles for HCC.

## Supporting Information

S1 Fig **miR-338-3p inhibitor has no effect on** ***HIF1A*** **3′UTR luciferase reporter activity.** Luciferase reporter assay of cells transfected with the *HIF1A* 3′UTR luciferase reporter plasmid with increasing amounts (20 to 50 nM) of NC or miR-338-3p-in (miR-338-3p-inhibitor in HCC cells two days post-transfection. Cells were cultured under hypoxia 24 h post-transfection; n = 4.
(TIF)

S2 Fig **miR-338-3p reduces HCC cell viability under normoxia.** Cell viability was determined by MTT assays in NC- or miR-338-3p- (50 nM) transfected HCC cells under normoxia conditions; n = 4.
(TIF)
