# Supplementary figures and images for: MiR-338-3p Inhibits Hepatocarcinoma Cells and Sensitizes These Cells to Sorafenib by Targeting Hypoxia-Induced Factor 1α
Source: PLoS One. 2014 Dec 22;9(12):e115565. doi: 10.1371/journal.pone.0115565 (PMC4274118; doi:10.1371/journal.pone.0115565)

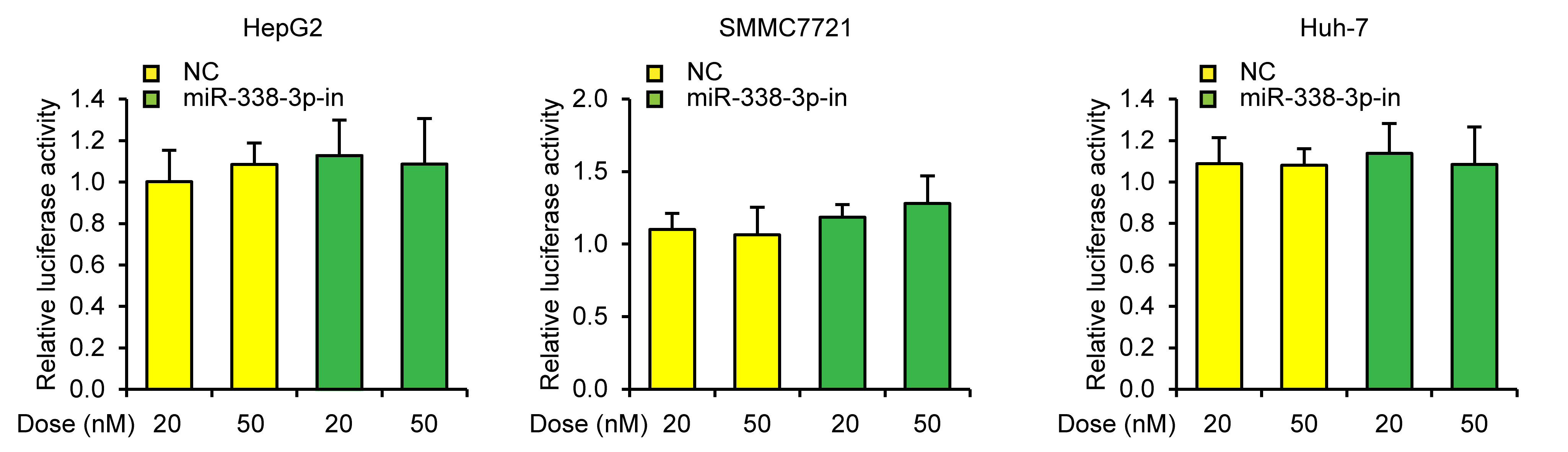

Supplement: S1 Fig — miR-338-3p inhibitor has no effect on HIF1A 3′UTR luciferase reporter activity. Luciferase reporter assay of cells transfected with the HIF1A 3′UTR luciferase reporter plasmid with increasing amounts (20 to 50 nM) of NC or miR-338-3p-in (miR-338-3p-inhibitor in HCC cells two days post-transfection. Cells were cultured under hypoxia 24 h post-transfection; n = 4. (TIF) [file pone.0115565.s001.tif]

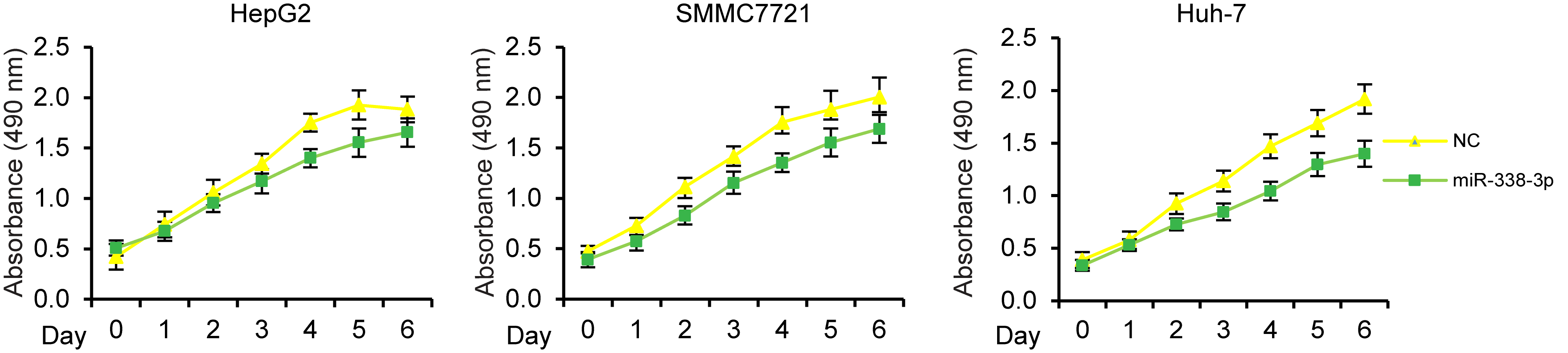

Supplement: S2 Fig — miR-338-3p reduces HCC cell viability under normoxia. Cell viability was determined by MTT assays in NC- or miR-338-3p- (50 nM) transfected HCC cells under normoxia conditions; n = 4. (TIF) [file pone.0115565.s002.tif]
